# Haplotype-based association analysis of general cognitive ability in Generation Scotland, the English Longitudinal Study of Ageing, and UK Biobank

**DOI:** 10.12688/wellcomeopenres.12171.1

**Published:** 2017-08-10

**Authors:** David M. Howard, Mark J. Adams, Toni-Kim Clarke, Eleanor M. Wigmore, Yanni Zeng, Saskia P. Hagenaars, Donald M. Lyall, Pippa A. Thomson, Kathryn L. Evans, David J. Porteous, Reka Nagy, Caroline Hayward, Chris S. Haley, Blair H. Smith, Alison D. Murray, G. David Batty, Ian J. Deary, Andrew M. McIntosh

**Affiliations:** 1 Division of Psychiatry, University of Edinburgh, Royal Edinburgh Hospital, Edinburgh, UK; 2 Medical Research Council Human Genetics Unit, Institute of Genetics and Molecular Medicine, University of Edinburgh, Edinburgh, UK; 3 Centre for Cognitive Ageing and Cognitive Epidemiology, University of Edinburgh, Edinburgh, UK; 4 Department of Psychology, University of Edinburgh, Edinburgh, UK; 5 Institute of Health and Wellbeing, University of Glasgow, Glasgow, UK; 6 Centre for Genomic and Experimental Medicine, Institute of Genetics and Molecular Medicine, University of Edinburgh, Edinburgh, UK; 7 Generation Scotland, Institute of Genetics and Molecular Medicine, University of Edinburgh, Edinburgh, UK; 8 Division of Population Health Sciences, University of Dundee, Dundee, UK; 9 Aberdeen Biomedical Imaging Centre, University of Aberdeen, Aberdeen, UK; 10 Department of Epidemiology and Public Health, University College London, London, UK

**Keywords:** haplotype association analysis, cognitive ability, intelligence, IQ

## Abstract

Background: Cognitive ability is a heritable trait with a polygenic architecture, for which several associated variants have been identified using genotype-based and candidate gene approaches. Haplotype-based analyses are a complementary technique that take phased genotype data into account, and potentially provide greater statistical power to detect lower frequency variants.

Methods: In the present analysis, three cohort studies (n _total_ = 48,002) were utilised: Generation Scotland: Scottish Family Health Study (GS:SFHS), the English Longitudinal Study of Ageing (ELSA), and the UK Biobank. A genome-wide haplotype-based meta-analysis of cognitive ability was performed, as well as a targeted meta-analysis of several gene coding regions.

Results: None of the assessed haplotypes provided evidence of a statistically significant association with cognitive ability in either the individual cohorts or the meta-analysis. Within the meta-analysis, the haplotype with the lowest observed *P*-value overlapped with the D-amino acid oxidase activator ( *DAOA*) gene coding region. This coding region has previously been associated with bipolar disorder, schizophrenia and Alzheimer’s disease, which have all been shown to impact upon cognitive ability. Another potentially interesting region highlighted within the current genome-wide association analysis (GS:SFHS: *P* = 4.09 x 10 ^-7^), was the butyrylcholinesterase ( *BCHE*) gene coding region. The protein encoded by *BCHE* has been shown to influence the progression of Alzheimer’s disease and its role in cognitive ability merits further investigation.

Conclusions: Although no evidence was found for any haplotypes with a statistically significant association with cognitive ability, our results did provide further evidence that the genetic variants contributing to the variance of cognitive ability are likely to be of small effect.

## Introduction

Cognitive ability facilitates the way in which we understand, interpret and interact with the world around us, and encompasses a broad range of neuropsychological skills, such as reasoning, various forms of memory, literacy, numeracy, logic, decision making, knowledge, and processing speed. There are positive correlations between each of these skills ^1^, and an individual’s aptitude for each skill can be quantified by completing specifically designed, validated and standardised tests. The results obtained using these tests are commonly combined to form an overall general cognitive function (‘ *g*’ or general intelligence) score. The heritability of *g* generally increases with age, with estimates ranging from 30 – 80% ^2, 3^. Several large, well-powered studies ^4– 8^ have reported a number of genome-wide significant associations for cognitive phenotypes using genotype data. Despite this, genotype-based analyses using single nucleotide polymorphism (SNP) data are unlikely to be able to fully capture the variation in the regions adjacent to the typed markers. This will be especially true for untyped or rare variants, or those variants that are in weak linkage disequilibrium (LD) with the SNPs found on common genotyping arrays. Haplotypes have the additional benefit of incorporating information from multiple variants where the DNA strand has been assigned.

Haplotype-based analyses of cognitive ability have focused on a number of specific gene coding regions: brain-derived neurotrophic factor ( *BDNF*) ^9, 10^, D-amino acid oxidase activator ( *DAOA*) ^11, 12^ and apolipoprotein E ( *APOE*) ^13, 14^. In the present analysis, these three regions will be assessed using the three available cohort studies, along with a genome-wide haplotype-based association analysis of cognitive ability. The Generation Scotland: Scottish Family Health Study (GS:SFHS) will be used as the discovery cohort, with the English Longitudinal Study of Ageing (ELSA) and UK Biobank used as replication cohort studies along with a meta-analyses of all three cohorts.

## Materials and methods

### Discovery cohort

***Generation Scotland: Scottish Family Health Study (GS:SFHS).*** GS:SFHS ^15, 16^ is a population and family-based cohort study of 23,960 individuals, of whom 20,195 were genotyped using the Illumina OmniExpress BeadChip (706,786 SNPs). Within GS:SFHS, there were 4,933 families containing at least two related individuals, including 1,799 families with two members, 1,216 families with three members and 829 families with four members, with the largest family containing 31 individuals. There were 1,789 individuals with no other family members in the cohort.

For quality control, individuals with a genotype call rate < 98% or who were identified as population outliers ^17^ through principal component analysis were removed, leaving 19,904 individuals. Quality control was also applied to the genomic data, with SNPs with a call rate < 98%, minor allele frequency (MAF) < 0.01 or that deviating from Hardy-Weinberg equilibrium ( *P* < 10 ^-6^) removed. This left a total of 561,125 autosomal SNPs.

### Replication cohorts

***English Longitudinal Study of Ageing (ELSA).*** ELSA ^18^ is a population-based cohort study consisting of 11,391 individuals, of which 7,597 were genotyped using the Illumina Omni 2.5–8 array (≈ 2.5M SNPs). SNPs which overlapped with the discovery sample were extracted, and individuals that reported a non-Caucasian ethnicity were removed to maximise homogeneity within the sample. This left 7,452 individuals with variant calls for 554,079 SNPs for analysis. There was no evidence of overlapping individuals between ELSA and GS:SFHS using a checksum-based approach, whereby a total of 500 randomly selected genome-wide SNPs, present across both cohort studies, were assigned to 10 equal-sized batches. A checksum was calculated using the cksum unix command for each individual and for each batch. If an individual in one cohort study had the same checksum for a specific batch as an individual in the other cohort, then this provided evidence of overlap between those two individuals (personal communication with Stephan Ripke).

***UK Biobank.*** UK Biobank ^19^ is a population-based cohort study consisting of 152,249 genotyped individuals with imputed genomic data for 72,355,667 variants ^20^. Individuals who reported a non-white British ethnicity or were identified as overlapping with either GS:SFHS (n = 174) or ELSA (n = 85), using the checksum-based approach described previously, were removed, leaving 119,832 individuals. Imputed variants with an infoscore ≥ 0.8, that were also genotyped in GS:SFHS, were extracted from the UK Biobank data, which identified 555,782 variants in common between the two cohorts.

### Genotype phasing and haplotype formation

Phasing of the genotype data within each cohort study was conducted using SHAPEIT v2.r837 ^21^. Genome-wide phasing was applied to the GS:SFHS discovery cohort. Within the replication cohort studies, phasing was conducted across a 50Mb window centred on haplotypes with *P* < 10 ^-6^ in the genome-wide analysis of the discovery cohort study, and the *BDNF*, *DAOA* and *APOE* gene coding regions. To improve phasing accuracy, the number of conditioning states per SNP was increased from the default of 100 states to 200 states. The default effective population size for European populations of 15,000 was used across the three cohorts. A 5Mb window size was used to conduct the phasing within GS:SFHS (rather than the default window size of 2Mb used for ELSA and UK Biobank), as this has been shown to be advantageous when larger amounts of identity by descent (IBD) sharing are present ^21^. The extensive family structure within GS:SFHS also meant the duoHMM method could be applied to that cohort. The duoHMM method combined the results of a MCMC algorithm with pedigree information to improve phasing accuracy ^22^. HapMap phase II b37 ^23^ was used to calculate the recombination rates between SNPs during phasing, and for the subsequent partitioning of the phased data into haplotypes.

Window sizes of 1cM, 0.5cM and 0.25cM were used to determine the SNPs included within each haplotype ^24^. A sliding window was used, sliding the window along a quarter of the respective window size. This produced a total of 97,333 windows with a mean number of SNPs per window of 157, 79 and 34 for the 1cM, 0.5cM and 0.25cM windows, respectively. The haplotype positions reported subsequently are given in base pair (bp) position (using GRCh37) and correspond to the outermost SNPs located within each haplotype. Those haplotypes containing less than 5 SNPs, or with a frequency < 0.005 or that deviating from Hardy-Weinberg equilibrium ( *P* < 10 ^-6^) were not assessed, but they were included as part of the alternative haplotype for the assessment of the remaining haplotypes. Following quality control there were 2,618,094 haplotypes for further analysis.

To estimate the correction required for multiple testing, the clump command within Plink v1.90 ^25^ was used to determine the number of independently segregating haplotypes. An LD r ^2^ threshold of 0.4 was used to classify a haplotype as independent and at this threshold there were 1,070,216 independently segregating haplotypes in the discovery cohort study. Therefore, a Bonferroni correction required that *P* < 5 × 10 ^-8^ for genome-wide significance. This was in alignment with the conventional level for significance used for sequence and SNP-based genome-wide association studies ^26^. Therefore in the present analysis, and for future genome-wide haplotype-based analyses using cohorts similar to GS:SFHS, the conventional *P*-value for significance can be applied.

### General cognitive ability

Within each cohort study, a principal component analysis was used to determine a general cognitive ability score ( *g*). This was calculated using the first unrotated principal component from the series of cognitive tests conducted within each cohort. The loadings used within each cohort are provided in Supplementary Table S1. The study demographics of each cohort for individuals for which *g* could be calculated are provided in Table 1. The GCTA-GREML ^27^ method was used to calculate SNP-based estimates for the heritability of *g*.

**Table 1.** Study demographics of Generation Scotland: Scottish Family Health Study (GS:SFHS), English Longitudinal Study of Ageing (ELSA) and UK Biobank for individuals with a general intelligence score.

	GS:SFHS	ELSA	UK Biobank
N	19,326	5,876	22,800
Males/Females	7,929/11,397	2,679/3,197	10,665/12,135
Age Range	18 – 94	31 – 90	40 – 75
Mean Age (s.dev.)	47.2 (14.9)	63.3 (9.4)	56.4 (7.7)

***Generation Scotland: Scottish Family Health Study (GS:SFHS).*** The following tests were used within GS:SFHS to calculate *g*: logical memory, verbal fluency, digit symbol-coding, and vocabulary. Logical memory was assessed using the Wechsler Memory Scale III ^28^. Verbal fluency was measured using a phonemic fluency test, requiring the participant to name as many words as possible beginning with a particular letter (C, F, and L were used) within a given timeframe ^29^. Digit symbol-coding was assessed using the Wechsler Adult Intelligence Scale III ^29^. Vocabulary was assessed using the Mill Hill Vocabulary Scale senior and junior synonyms combined ^30^. Additional information regarding the cognitive ability variables available within GS:SFHS has been published previously ^14, 15, 31^. *g* explained 0.43 of the variance across the four tests and was available for 19,326 individuals.

***English Longitudinal Study of Ageing (ELSA).*** The first wave of the cognitive tests conducted by ELSA were used to calculate *g* for this cohort: processing speed, verbal memory and verbal fluency. Processing speed was calculated using a letter cancellation task with participants searching a large grid of letters for the letters P and W and crossing those out. Verbal memory was assessed using a ten-word list-learning task. Verbal fluency was measured by the number of different animal species that could be named in one minute. Further information regarding these cognitive tests is provided elsewhere ^32, 33^. There were 5,876 individuals for which *g* could be calculated, with *g* explaining 0.49 of the total variance across the three cognitive tests.

***UK Biobank.*** The touchscreen cognitive tests conducted as part of the online follow-up within UK Biobank were used to derive *g*. Some of these tests have yet to be reported elsewhere and are therefore covered in greater detail here. The following tests were used within this cohort study: fluid intelligence test (UK Biobank Field 20191), trail making test (mean of UK Biobank Fields 20156 and 20157), symbol digit substitution test (UK Biobank Field 20159) and numeric memory test (UK Biobank Field 20240). The fluid intelligence test consisted of 13 multiple-choice questions to be answered within two minutes, with a score based on the number of correct answers. For the trail making test participants were firstly presented with a screen containing a series of numbers from 1 to 25, each contained within a circle. Starting with the circle containing the number 1, the participants then had to use the computer mouse to click on the numbers in ascending order. Secondly, the participants were presented with circles containing the numbers 1 to 13 and the letters A to L. For this test the participants had to click the circles in the order 1, A, 2, B, 3, C, 4, D, etc. For both the trail making tests the time taken to complete each test was recorded, with the log of the mean time across the two tests taken as the final score for this test. The symbol digit coding test consisted of a series of eight symbols that corresponded to eight numbers. The participants were then repetitively presented with eight symbols in a specific order that required recoding to their numerical equivalents. The number of correctly recoded sequences within one minute was recorded. The numeric memory test began with a two-digit number being presented, after a short delay the participant was then required to enter the number presented. The length of the number presented was then incremented by one digit each time with the participant required to recall the full number correctly, up to a maximum of 12 digits. The maximum number of digits recalled successfully was recorded. The proportion of variance explained by *g* across the four tests was 0.51 and was available for 22,800 individuals. The proportion of variance explained by *g* within the online follow-up was greater than that reported (≈ 0.4) by Lyall, Cullen ^34^ for the original cognitive tests conducted within UK Biobank.

### Statistical analysis

***Discovery cohort.*** A genome-wide haplotype-based association analysis was conducted within GS:SFHS using a mixed linear model within GCTA v1.25.0 ^35^:

**y** = **Xβ** + **Z**_1_ **u** + **Z**_2_ **v** + **ε**

where **y** was the vector of observations for *g*. **β** was the matrix of fixed effects, including haplotype, sex and age. A SNP-based genomic relationship matrix ^27^ ( **G**) using the ‘leave one chromosome out’ methodology ^35^, which excluded the chromosome of the assessed haplotype, was fitted as a random effect, **u**, taking into account the genomic relationships as MVN (0, $\text{G}\sigma_{\text{u}}^{2}$). **v** was a random effect fitting a second genomic relationship matrix **G _t_** as MVN (0, ${\text{G}}_{\text{t}}\sigma_{\text{v}}^{2}$), which modelled only the more closely related individuals ^36^. **G _t_** was identical to **G**, except that off-diagonal elements < 0.05 were set to 0. **X**, **Z**_1_ and **Z**_2_ were the corresponding incidence matrices. **ε** was the vector of residual effects and was assumed to be normally distributed as MVN (0, **I** $\sigma_{\varepsilon }^{2}$).

GS:SFHS is a family-based cohort and therefore LD score regression ^37^ was used to test for the existence of population stratification by examining the summary statistics obtained from the above mixed model. The fitting of a single genomic relationship matrix, **G**, provided evidence of significant population stratification (intercept = 1.051 ± 0.004). Whilst the simultaneous fitting of the matrices **G** and to **G _t_** together produced no evidence of population stratification (intercept = 0.998 ± 0.003), hence the fitting of two matrices for GS:SFHS.

***Replication cohorts.*** A mixed linear model was used to assess the haplotypes in ELSA and UK Biobank which were identified in the GS:SFHS discovery cohort study with *P* < 10 ^-6^ and those haplotypes in GS:SFHS that overlapped with the *BDNF*, *DAOA* and *APOE* gene coding regions. This was conducted using GCTA v1.25.0 ^35^:

**y** = **Xβ** + **Z**_1_ **u** + **ε**

where **y** was the vector of binary observations for g. **β** was the matrix of fixed effects, including haplotype, sex and age, and for UK Biobank, genotyping batch and recruitment centre were also fitted. **u** was fitted as a random effect taking into account the SNP-based genomic relationships as MVN (0, $\text{G}\sigma_{\text{u}}^{2}$) and also implemented the ‘leave one chromosome out’ methodology ^35^. **X** and **Z _1_** were the corresponding incidence matrices and **ε** was the vector of residual effects and was assumed to be normally distributed as MVN (0, **I** $\sigma_{\varepsilon }^{2}$). Replication success was judged on the statistical significance of each haplotype using an inverse variance-weighted meta-analysis across all three cohorts conducted with Metal ^38^.

## Results

**Figure 1.**
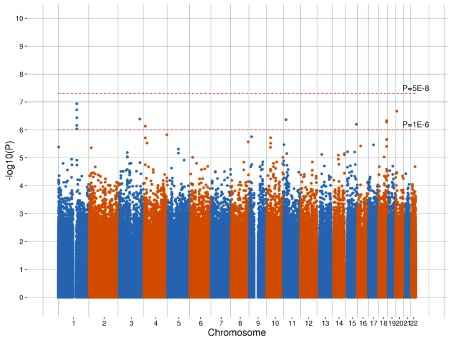
Manhattan plot representing the –log _10_ *P*-values for an association between each assessed haplotype and cognitive score in the Generation Scotland: Scottish Family Health Study cohort study.

A genome-wide haplotype-based association analysis for general cognitive ability, using a principal component derived measure of *g*, was conducted using 2,618,094 haplotypes within the GS:SFHS discovery cohort study. A genome-wide Manhattan plot of –log _10_ *P*-values is provided in Figure 1, with a q-q plot provided in Supplementary Figure S1. No haplotypes exceeded the genome-wide significance threshold ( *P* < 5 × 10 ^-8^) for an association with *g*. Within the discovery cohort study, 12 haplotypes had *P* < 10 ^-6^, and replication was sought for these 12 haplotypes within ELSA and UK Biobank. Summary statistics regarding each cohort study and the meta-analysis of these haplotypes (after applying an LD r ^2^ threshold of 0.4 to identify those that are independently segregating) are provided in Table 2. The frequencies of the haplotypes within each cohort, for the seven independently segregating haplotypes with *P* < 10 ^-6^ in the discovery cohort, along with the protein coding genes that these haplotypes overlapped, are provided in Supplementary Table S2.

**Table 2.** Independently segregating (linkage disequilibrium r ^2^ threshold of 0.4) haplotypes sorted by *P*-value obtained in the meta-analysis and with a *P*-value < 10 ^-6^ for an association with cognitive ability within the discovery cohort study, Generation Scotland: Scottish Family Health Study (GS:SFHS).

Haplotype		GS:SFHS		ELSA		UK Biobank		Meta-analysis	
Chr	Position (bp)	Beta (s.e.)	*P*-value	Beta (s.e.)	*P*-value	Beta (s.e.)	*P*-value	Direction	*P*-value
18	64252341 - 64568113	0.23 (0.05)	5.21 × 10 ^-7^	0.08 (0.06)	0.17	0.01 (0.02)	0.54	+++	0.001
3	165337109 - 166522847	0.60 (0.12)	4.09 × 10 ^-7^	-0.02 (0.15)	0.9	0.06 (0.06)	0.36	+-+	0.003
20	9288522 - 9726640	0.55 (0.11)	2.13 × 10 ^-7^	-0.003 (0.06)	0.96	0.04 (0.05)	0.38	+-+	0.008
1	150165849 - 151140732	0.51 (0.10)	9.20 × 10 ^-7^	0.12 (0.14)	0.37	0.002 (0.06)	0.97	+++	0.01
4	11448182 - 11547967	0.32 (0.06)	7.36 × 10 ^-7^	0.11 (0.07)	0.96	0.01 (0.03)	0.83	+++	0.04
11	20184958 - 20297638	-0.56 (0.11)	4.31 × 10 ^-7^	-0.03 (0.13)	0.84	0.12 (0.06)	0.04	--+	0.6
15	94701431 - 94729657	-0.27 (0.05)	6.33 × 10 ^-7^	-0.10 (0.07)	0.14	0.02 (0.01)	0.16	--+	0.85

Beta values, standard errors and *P*-values are given for GS:SFHS, English Longitudinal Study of Ageing (ELSA), UK Biobank and a meta-analysis of all three cohort studies. Genomic location is determined by position on the GRCh37 assembly.

Of the 12 haplotypes with *P* < 10 ^-6^ in GS:SFHS, none were nominally significant ( *P* ≥ 0.05) in ELSA. Within UK Biobank the only haplotype to be nominally significantly ( *P* < 0.05) associated with *g* was located on chromosome 11 and this was in the opposite direction to that observed for GS:SFHS. The smallest *P*-value (1.46 × 10 ^-3^) observed within the genome-wide meta-analysis was located on chromosome 18 and although neither of the replication cohort studies were nominally significant, their effects were in the same direction as that observed within GS:SFHS. The genetic variance explained by each of the haplotypes within GS:SFHS was small, ranging from 3.93 × 10 ^-3^ – 4.63 × 10 ^-3^. A power analysis revealed that the sample sizes for the replication cohorts were large enough to provide statistical power in excess of 0.99, assuming an effect size equivalent to that observed in the discovery cohort study.

The SNP-based heritability of *g* was calculated using GCTA-GREML ^27^ and was 0.41 (s.e = 0.05) for GS:SFHS, 0.17 (s.e. = 0.06) for ELSA, and 0.21 (s.e. = 0.02) for UK Biobank. The heritability of *g* within GS:SFHS was calculated using an unrelated subsample of 7 388 individuals, whereby one of a pair of individuals was removed if they shared a genotype-based relatedness of > 0.025.

## *BDNF, DAOA* and *APOE* gene coding regions

None of the haplotypes overlapping the *BDNF, DAOA* and *APOE* gene coding regions were statistically significant at the genome-wide level (P ≥ 5 × 10 ^-8^) in the meta-analysis or in the single cohort analyses. The top five independently segregating haplotypes (following the application of an LD r ^2^ threshold of 0.4) in terms of statistical significance achieved in the meta-analysis for each of the gene coding regions are shown in Table 3. There were 214 haplotypes that overlapped the *BDNF* gene coding region and the lowest *P*-value obtained in the meta-analysis was 1.35 × 10 ^-3^ for a haplotype with a positive effect (beta = 0.31 ± 0.10) on *g*. The *DAOA* gene coding region overlapped with 410 assessed haplotypes, with the lowest *P*-value = 1.53 × 10 ^-5^ within the meta-analysis for a haplotype with a positive effect (beta = 0.20 ± 0.05) on *g*. Overlapping the *APOE* gene coding region there were 325 assessed haplotypes, of which the lowest observed *P*-value in the meta-analysis was 7.50 × 10 ^-4^ for a haplotype with a positive effect (beta = 0.18 ± 0.05).

**Table 3.** Independently segregating (linkage disequilibrium r ^2^ threshold of 0.4) haplotypes overlapping the brain-derived neurotrophic factor (BDNF), D-amino acid oxidase activator (DAOA) and apolipoprotein E (APOE) gene coding regions.

Haplotype		GS:SFHS		ELSA		UK Biobank		Meta-analysis	
Gene	Chr:Position (bp)	Beta (s.e.)	*P*-value	Beta (s.e.)	*P*-value	Beta (s.e.)	*P*-value	Direction	*P*-value
*BDNF*	11:27337843-27778592	0.34 (0.12)	0.007	0.27 (0.15)	0.08	na	na	++?	0.001
	11:27444517-27787783	0.31 (0.12)	0.01	0.24 (0.15)	0.11	na	na	++?	0.003
	11:27337843-27778592	0.22 (0.09)	0.01	0.10 (0.09)	0.27	na	na	++?	0.009
	11:27662826-27990119	0.25 (0.09)	0.006	0.07 (0.10)	0.47	na	na	++?	0.01
	11:27020461-27749725	-0.28 (0.11)	0.01	-0.10 (0.10)	0.31	na	na	--?	0.02
*DAOA*	13:106140780-106393146	0.25 (0.11)	0.03	0.16 (0.14)	0.23	0.20 (0.06)	3.54 × 10 ^-4^	+++	1.53 × 10 ^-5^
	13:106098389-106240125	0.28 (0.11)	0.009	0.08 (0.11)	0.47	0.13 (0.05)	0.005	+++	2.63 × 10 ^-4^
	13:106140780-106240125	0.22 (0.10)	0.02	0.08 (0.10)	0.42	0.12 (0.04)	0.005	+++	4.03 × 10 ^-4^
	13:106066286-106154577	-0.22 (0.12)	0.07	-0.06 (0.14)	0.67	-0.22 (0.07)	0.002	---	5.91 × 10 ^-4^
	13:106065361-106133365	-0.18 (0.11)	0.12	-0.04 (0.13)	0.75	-0.20 (0.06)	0.001	---	6.50 × 10 ^-4^
*APOE*	19:45290685-45422561	0.28 (0.11)	0.009	0.18 (0.13)	0.16	0.14 (0.07)	0.05	+++	7.50 × 10 ^-4^
	19:45318153-45422561	0.27 (0.09)	0.003	0.20 (0.10)	0.05	0.06 (0.05)	0.28	+++	0.002
	19:45389224-45548502	0.14 (0.08)	0.07	0.11 (0.09)	0.21	0.08 (0.04)	0.04	+++	0.004
	19:45390685-45422561	0.09 (0.13)	0.45	-0.15 (0.13)	0.26	-0.17 (0.06)	0.004	+--	0.01
	19:45351746-45422561	0.39 (0.10)	1.24 × 10 ^-4^	-0.09 (0.11)	0.4	0.08 (0.05)	0.14	+-+	0.01

Beta values, standard errors and *P*-values are given for Generation Scotland: Scottish Family Health Study (GS:SFHS), English Longitudinal Study of Ageing (ELSA), UK Biobank and a meta-analysis of all three cohort studies. There were no UK Biobank individuals that carried the shown BDNF overlapping haplotypes. Haplotypes are sorted by *P*-value obtained in the meta-analysis within each gene coding region. Genomic location is determined by position on the GRCh37 assembly.

## Discussion

Twelve haplotypes were identified in the GS:SFHS discovery cohort study with a *P*-value ** < 10 ^-6^ for an association with *g*, although none of these reached genome-wide significance ( *P* > 5 × 10 ^-8^). Replication of these twelve haplotypes was sought and not found within the ELSA and UK Biobank cohort studies. Both of these cohorts were sufficiently powered cohorts to detect effects of the sizes observed within GS:SFHS, assuming that the haplotypes were in linkage equilibrium with the causal variant. Therefore, despite SNP-based heritability estimates ranging from 0.17 to 0.41 for *g* across the three cohort studies, there was no evidence for any haplotypes significantly associated with cognitive ability.

The haplotypes with *P* < 10 ^-6^ within the discovery cohort study overlapped with a number of gene coding regions. In terms of biological viability the most notable of these haplotypes was located on chromosome 3 that overlapped with the coding region for the butyrylcholinesterase ( *BCHE*) gene. *BCHE* has been shown to have a role in cognitive ability within humans ^39, 40^ as well as rodents ^41, 42^. SNP variants close to this coding region, which overlapped with the haplotype on chromosome 3, have also been shown to be significantly associated ( *P* = 2.69 × 10 ^−8^) with the cortical deposition of amyloid-β peptide ^43^. This deposition is thought to be an initiating factor in the pathology of Alzheimer's disease ^44, 45^, which has a known impact on cognitive ability. Furthermore, the *BCHE*-K variant (rs1803274) has been shown to have an effect on the progression of Alzheimer’s disease ^46, 47^ and an interaction with the *APOE* ε4 allele among those with late-onset of the disease ^48^. The *BCHE*-K variant was not genotyped within GS:SFHS but it is located within the bounds of the haplotype on chromosome 3. This haplotype was analysed and not found to be associated with Alzheimer’s disease ( *P* ≥ 0.05) within GS:SFHS, using the same mixed linear model described previously and self-declared Alzheimer’s disease as the phenotype. However, the prevalence of the disease in this cohort (0.14%) is likely to have limited the power to detect an effect.

The targeted meta-analyses of the *BDNF*, *DAOA* and *APOE* gene coding regions did not provide evidence of genome-wide significant haplotypes ( *P* ≥ 5 × 10 ^−8^) associated with cognitive ability. The *BDNF* region yielded several haplotypes which were more statistically significant than those found by Wilkosc, Szalkowska ^9^ or Warburton, Miyajima ^10^. *BDNF* is involved in the development of synaptic connectivity in the central nervous system ^49^ and therefore represents a potential source of cognitive score variance. The most significant haplotype ( *P* = 1.53 × 10 ^-5^) identified across all meta-analyses was in the *DAOA* coding region. SNP variants located within the *DAOA* gene have also been associated with diseases related to the brain: bipolar disorder ^50^, Alzheimer’s disease ^51^ and, potentially, schizophrenia ^52^. These diseases are known to be associated with decrements in cognitive ability. Haplotypes within the *APOE* gene coding region have been studied previously within GS:SFHS ^14^, although the haplotypes examined previously were considerably shorter, formed of two variants and used the cognitive tests individually rather than forming an overall *g* score. The *P*-value of the most significant haplotype in the *APOE* region in the present analysis was stronger than the haplotypes assessed by Marioni, Campbell ^14^, but was not genome-wide significant ( *P* ≥ 5 × 10 ^−8^).

The cohort studies selected for analysis should be relatively homogenous, as they are a subset of the British population, this can be observed by the consistency of the haplotype frequencies shown in Supplementary Table S2. However, there were some differences in the cognitive tests applied between the studies. The size of the present analysis is comparable number to that of the genotyped-based genome-wide association study of cognitive ability conducted by the CHARGE consortium ^4^. Their paper drew the conclusion that there were likely to be many genes of small effect contributing to the genetic variance underlying cognitive ability. Based on the observed heritability of the trait, but a lack of genome-wide significant haplotypes in the present analyses, this conclusion continues to hold true.

## Conclusions

None of the haplotypes analysed in this study achieved genome-wide significance ( *P* ≥ 5 × 10 ^−8^) for an association with cognitive ability within any of the cohort studies, or in the meta-analysis. The genome-wide analysis identified a haplotype within the *BCHE* gene coding region which may play a role in cognitive ability and this warrants further analysis. Although haplotypes should allow the detection of signals from rarer causal variants compared to a typical genotype-based analysis, there was no evidence for genome-wide significant haplotypes for the window sizes tested. Potentially shorter and therefore more common haplotypes could be assessed, however to detect rarer genetic contributions to highly polygenic traits such as cognitive ability, there remains a requirement for larger sample sizes.

## Data availability

The data referenced by this article are under copyright with the following copyright statement: Copyright: © 2017 Howard DM et al.

Due to the confidential nature of the genetic data and cognitive test scores of participants, it is not possible to publically share the data on which our analysis was based. Generation Scotland (GS) data is available on request to: access@generationscotland.org, with further information available from http://www.ed.ac.uk/generation-scotland. Each application requires the completion of a data and materials transfer agreement, the conditions of which be determined on a case by case basis. GS has Research Tissue Bank status, and the GS Access Committee reviews applications to ensure that they comply with legal requirements, ethics and participant consent. UK Biobank data is available for health related research on request to: access@ukbiobank.ac.uk, with further information relating to data access available from http://www.ukbiobank.ac.uk/register-apply. The English Longitudinal Study of Ageing data is available on request to: n.rogers@ucl.ac.uk, with further information regarding data access available from https://www.elsa-project.ac.uk.

## Ethical statement

Ethics approval for the Generation Scotland study was given by the NHS Tayside committee on research ethics (reference 05/S1401/8). The UK Biobank study was conducted under generic approval from the NHS National Research Ethics Service (approval letter dated 17th June 2011, Ref 11/NW/0382). Ethical approval for the English Longitudinal Study of Ageing was obtained from the London Multi-Centre Research Ethics Committee. All participants gave full informed written consent to participate within each study.

## Supplementary material

**Supplementary Table S1.** Loadings used for each of the cognitive tests to calculate the general intelligence ( *g*) score within Generation Scotland: Scottish Family Health Study (GS:SFHS), the English Longitudinal Study of Ageing (ELSA) and UK Biobank.

**Supplementary Table S2.** Haplotype frequency and overlap with gene coding regions of the independently segregating (linkage disequilibrium r ^2^ threshold of 0.4) haplotypes with a *P*-value < 10 ^-6^ for an association with cognitive ability within the discovery cohort study, Generation Scotland: Scottish Family Health Study (GS:SFHS). Haplotype frequencies are also provided for the English Longitudinal Study of Ageing (ELSA) and UK Biobank cohort studies. Haplotypes are sorted by chromosome with genomic locations determined by position on the GRCh37 assembly.

**Supplementary Figure S1.** Q-Q plot representing the observed –log _10_ *P*-values against the expected –log _10_ *P*-values for an association between each assessed haplotype and cognitive score in the Generation Scotland: Scottish Family Health Study cohort study.
