# Supplementary material for: Haplotype-based association analysis of general cognitive ability in Generation Scotland, the English Longitudinal Study of Ageing, and UK Biobank
Source: Wellcome Open Res. 2017 Aug 10;2:61. [Version 1] doi: 10.12688/wellcomeopenres.12171.1 (PMC5605947; doi:10.12688/wellcomeopenres.12171.1)
Supplement: Supplementary file 1 [file wellcomeopenres-2-13175-s0000.tgz › ebac1a31-e47e-4c5c-9997-2c242f90cf65.pdf]

Supplementary Table S1. Loadings used for each of the cognitive tests to calculate the general intelligence (g) score within Generation Scotland: Scottish Family Health Study (GS:SFHS), the English Longitudinal Study of Ageing (ELSA) and UK Biobank.

GS:SFHS		ELSA		UK Biobank	
**Test**	**Loading**	**Test**	**Loading**	**Test**	**Loading**
Logical memory	0.64	Processing speed	0.42	Fluid intelligence	0.68
Verbal fluency	0.71	Verbal memory	0.63	Trail making	-0.81
Digit symbol-coding	0.61	Verbal fluency	0.65	Symbol digit substitution	0.77
Vocabulary	0.67			Numeric memory	0.56
