# Supplementary material for: Haplotype-based association analysis of general cognitive ability in Generation Scotland, the English Longitudinal Study of Ageing, and UK Biobank
Source: Wellcome Open Res. 2017 Aug 10;2:61. [Version 1] doi: 10.12688/wellcomeopenres.12171.1 (PMC5605947; doi:10.12688/wellcomeopenres.12171.1)
Supplement: Supplementary file 2 [file wellcomeopenres-2-13175-s0001.tgz › 44538e3c-9019-43e6-9332-226319a03127.pdf]

Supplementary Table S2. Haplotype frequency and overlap with gene coding regions of the independently segregating (linkage disequilibrium r2 threshold of 0.4) haplotypes with a P-value < 10-6 for an association with cognitive ability within the discovery cohort study, Generation Scotland: Scottish Family Health Study (GS:SFHS). Haplotype frequencies are also provided for the English Longitudinal Study of Ageing (ELSA) and UK Biobank cohort studies. Haplotypes are sorted by chromosome with genomic locations determined by position on the GRCh37 assembly.

		**Haplotype Frequency**			
**Chr**	**Position (bp)**	**GS:SFHS**	**ELSA**	**UK Biobank**	**Protein coding genes located along haplotype**
1	150165849 - 151140732	0.008	0.006	0.006	ANP32E, CA14, APH1A, C1orf54, C1orf51, MRPS21, PRPF3, RPRD2, TARS2, ECM1, ADAMTSL4, MCL1, ENSA, GOLPH3L, HORMAD1, CTSS, CTSK, ARNT, SETDB1, CERS2, ANXA9, FAM63A, PRUNE, BNIPL, C1orf56, CDC42SE1, MLLT11, GABPB2, SEMA6C
3	165337109 - 166522847	0.005	0.005	0.005	BCHE
4	11448182 - 11547967	0.02	0.026	0.024	
11	20184958 - 20297638	0.007	0.006	0.006	
15	94701431 - 94729657	0.028	0.023	0.102	
18	64252341 - 64568113	0.04	0.034	0.033	CDH19
20	9288522 - 9726640	0.007	0.009	0.009	PLCB4, LAMP5, PAK7
