# Supplementary figures and images for: Haplotype-based association analysis of general cognitive ability in Generation Scotland, the English Longitudinal Study of Ageing, and UK Biobank
Source: Wellcome Open Res. 2017 Aug 10;2:61. [Version 1] doi: 10.12688/wellcomeopenres.12171.1 (PMC5605947; doi:10.12688/wellcomeopenres.12171.1)

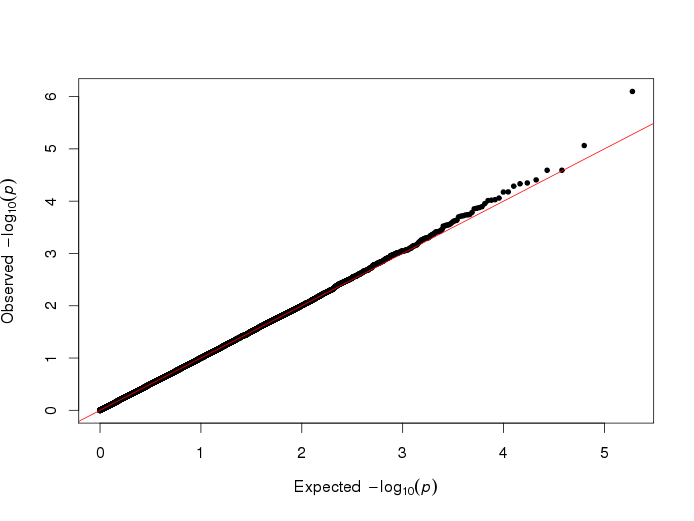

Supplement: Supplementary file 3 [file wellcomeopenres-2-13175-s0002.tgz › 3547fa91-ffb9-48c2-bfcf-f240bf15935e.tif]
