# Supplementary material for: Haplotype-based association analysis of general cognitive ability in Generation Scotland, the English Longitudinal Study of Ageing, and UK Biobank
Source: Wellcome Open Res. 2017 Aug 10;2:61. [Version 1] doi: 10.12688/wellcomeopenres.12171.1 (PMC5605947; doi:10.12688/wellcomeopenres.12171.1)
Supplement: Peer review history

# Peer review: Referee response for version 1

**DOI:** 10.21956/wellcomeopenres.13175.r26013

**Published:** 2017-09-15

**Authors:** Heiner Rindermann

**Affiliations:** 1 Department of Psychology, Chemnitz University of Technology, Chemnitz, Germany

The paper tries to find genetic associations with intelligence. For this purpose, haplotypes and intelligence g factors are correlated at the individual level using three large British data sets. The study could not find significant (statistically significant, theoretically important) correlations between genomes and intelligence.

 Study design, analysis etc. are well done.

 I have three suggestions:

 First, present your results also using easy to understand effect sizes as:

1. average correlations between certain genes/haplotypes and intelligence and
2. IQ effects in the IQ scale. If there are zero-correlations present them. If there are zero-IQ-effects present them.

Second, describe how evolutionarily diverse your samples are – are there only British people or also people from Northern Africa, Middle East, Central Asia and Sub-Saharan Africa?

 Third, there are theoretically important and empirically found associations between genes (being coding or not) and intelligence at the individual and group level, e.g.: Davis *et al*, (2015), Piffer (2013), Piffer (2015) and Rindermann *et al.*(2012).

 In these studies were mentioned several genes, genetic markers and haplogroups, e.g. DUF1220, COMT Val158Met and the haplogroups I, R1a, R1b, N, J1, E, T[+L], being associated to intelligence.

 Do you have data on this? Can you replicate the findings?

I have read this submission. I believe that I have an appropriate level of expertise to confirm that it is of an acceptable scientific standard.

## References

[1] DavisJMSearlesVBAndersonNKeeneyJRaznahanAHorwoodLJFergussonDMKennedyMAGieddJSikelaJM: DUF1220 copy number is linearly associated with increased cognitive function as measured by total IQ and mathematical aptitude scores. *Hum Genet*.2015;134(1) : 10.1007/s00439-014-1489-2 67-75 10.1007/s00439-014-1489-2 25287832PMC5898241

[2] PifferD.: Correlation of the COMT Val158Met polymorphism with latitude and a hunter-gather lifestyle suggests culture–gene coevolution and selective pressure on cognition genes due to climate. *Anthropological Science*.2013;121(3) : http://doi.org/10.1537/ase.130731 161-171 10.1537/ase.130731

[3] PifferD.: A review of intelligence GWAS hits: Their relationship to country IQ and the issue of spatial autocorrelation. *Intelligence*.2015;53: https://doi.org/10.1016/j.intell.2015.08.008 43-50 10.1016/j.intell.2015.08.008

[4] RindermannHWoodleyM.A.StratfordJ.: Haplogroups as evolutionary markers of cognitive ability. *Intelligence*.2012;40(4) : https://doi.org/10.1016/j.intell.2012.04.002 362-375 10.1016/j.intell.2012.04.002

# Peer review: Referee response for version 1

**DOI:** 10.21956/wellcomeopenres.13175.r25393

**Published:** 2017-09-08

**Authors:** Antony Payton

**Affiliations:** 1 Centre for Epidemiology, Division of Population Health, Health Services Research and Primary Care, University of Manchester, Manchester, UK

Abstract

**“Cognitive ability is a heritable trait with a polygenic architecture, for which several associated variants have been identified using genotype-based and candidate gene approaches”**

 A recent GWAS by Sniekers *et al* identified 336 variants in 22 genes associated with human intelligence. This is more than “several”.

 Genotyping is used in candidate gene approaches. Does the author mean GWAS and candidate gene approaches?

**“Haplotype-based analyses are a complementary technique that take phased genotype data into account, and potentially provide greater statistical power to detect lower frequency variants.”**

 Grammar needs checking.

 Introduction

**“Haplotypes have the additional benefit of incorporating information from multiple** **variants where the DNA strand has been assigned.”**

 What does “where the DNA strand has been assigned” mean? This is too vague.

I have read this submission. I believe that I have an appropriate level of expertise to confirm that it is of an acceptable scientific standard.

## References

[1] SniekersSStringerSWatanabeKJansenPRColemanJRIKrapohlETaskesenEHammerschlagAROkbayAZabanehDAminNBreenGCesariniDChabrisCFIaconoWGIkramMAJohannessonMKoellingerPLeeJJMagnussonPKEMcGueMMillerMBOllierWERPaytonAPendletonNPlominRRietveldCATiemeierHvan DuijnCMPosthumaD: Genome-wide association meta-analysis of 78,308 individuals identifies new loci and genes influencing human intelligence. *Nat Genet*.2017;49(7) : 10.1038/ng.3869 1107-1112 10.1038/ng.3869 28530673PMC5665562
